# The *opgC* gene is required for OPGs succinylation and is osmoregulated through RcsCDB and EnvZ/OmpR in the phytopathogen *Dickeya dadantii*

**DOI:** 10.1038/srep19619

**Published:** 2016-01-21

**Authors:** Sébastien Bontemps-Gallo, Edwige Madec, Catherine Robbe-Masselot, Erika Souche, Jacqueline Dondeyne, Jean-Marie Lacroix

**Affiliations:** 1 Structural and Functional Glycobiology Unit, UMR CNRS-Lille1 8576, University of Lille, 59655 Villeneuve d’Ascq cedex, France; 2 Center for Human Genetics, University of Leuven, Herestraat 49, 3000 Leuven, Belgium

## Abstract

Osmoregulated periplasmic glucans (OPGs) are a family of periplasmic oligosaccharides found in the envelope of most Proteobacteria. They are required for virulence of zoo- and phyto-pathogens. The glucose backbone of OPGs is substituted by various kinds of molecules depending on the species, *O*-succinyl residues being the most widely distributed. In our model, *Dickeya dadantii*, a phytopathogenic bacteria causing soft rot disease in a wide range of plant species, the backbone of OPGs is substituted by *O*-succinyl residues in media of high osmolarity and by *O*-acetyl residues whatever the osmolarity. The *opgC* gene encoding a transmembrane protein required for the succinylation of the OPGs in *D. dadantii* was found after an *in silico* search of a gene encoding a protein with the main characteristics recovered in the two previously characterized OpgC of *E. coli* and *R. sphaeroides, i.e.* 10 transmembrane segments and one acyl-transferase domain. Characterization of the *opgC* gene revealed that high osmolarity expression of the succinyl transferase is controlled by both the EnvZ-OmpR and RcsCDB phosphorelay systems. The loss of *O*-succinyl residue did not affect the virulence of *D. dadantii,* suggesting that only the glucose backbone of OPGs is required for virulence.

*Dickeya dadantii* is a phytopathogenic Enterobacteria that causes soft rot disease in a wide range of plant species, including crops^1^. *Dickeya spp*. are directly responsible for from 5 to 25% of the potato production losses in Europe and Israel^2^. The pathogen is listed as an A2 quarantine organism by the European and Mediterranean Plant Protection Organization^3^,^4^,^5^. Maceration, a visible symptom of the disease, is the result of the synthesis and secretion of a set of plant cell-wall-degrading enzymes particularly pectinases^6^,^7^. However, numerous additional factors, such as osmoregulated periplasmic glucans (OPGs), are required for full virulence.

OPGs are important intrinsic components of the cell envelope and are required for virulence and symbiosis of Proteobacteria^8^. Their common features are: their abundance decreases as the osmolarity of the medium increases, the fact that glucose is the sole sugar mainly joined by β-linkages. They can be substituted with various molecules depending on the species^9^. In Enterobacteria, the backbone consists of linear β-1,2 linked glucose residues branched by β-1,6 linked glucose residues synthesized by the *opgGH* operon products. OpgH is a transmembrane glucosyl transferase catalyzing the linear β-1,2 glucose backbone from UDP-glucose in the presence of ACP. The periplasmic OpgG glucosyl-transferase branch β-1,6 glucose residues from this growing linear backbone and liberate OPGs, ranging from 4 to 12 glucose residues, into the periplasm. During elongation, this glucose backbone is substituted by various kinds of molecules^9^,^10^. In *D. dadantii*, the glucose backbone of OPGs is substituted by *O*-acetyl and *O-*succinyl residues. OPGs are constitutively substituted by *O*-acetyl residues while *O-*succinyl residues are conditionally associated with OPGs since succinylation occurs only in media of high osmolarity^11^.

In *D. dadantii*, *opgG* and *opgH* mutant strains are completely devoid of OPGs and exhibit pleiotropic phenotypes including a total loss of virulence, loss of motility, increased synthesis of exopolysaccharides and induction of a general stress response indicating impairment in the perception of the environment^12^,^13^. Most of these phenotypic characteristics are the result of the requirement of OPGs to achieve a low level of activation of the RcsCDB two-component signalling pathways^14^,^15^,^16^.

Two-component systems (also called phosphorelays) are the key to gene expression plasticity in response to environmental variations. The paradigm of the two-component systems is the EnvZ/OmpR system of *Escherichia coli*. Under increasing osmolarity, a sensor histidine kinase, EnvZ, autophosphorylates and transfers its phosphate group to its cognate regulator, OmpR, which in turn regulates the expression of different target genes involved in the osmotic stress responses^17^. Actually, the only known role of EnvZ/OmpR in *D. dadantii* is the regulation of two oligogalacturonate outer membrane channels^18^.

The RcsCDB phosphorelay is restricted to Enterobacteria. Under various stimuli, one of them being increasing osmolarity, RcsC, the transmembrane kinase, phosphorylates the RcsB cytoplasmic regulator via the transmembrane protein RcsD. In several Enterobacteria, such as *D. dadantii*, it was shown that activation of the RcsCDB system enhanced exopolysaccharide synthesis, cell division and decreased virulence and motility^15^,^19^,^20^,^21^.

The following study was conducted to identify the gene responsible for the succinylation of the OPGs in *D. dadantii* and the regulation of its expression. We show that the regulation of this gene by osmolarity variation occurs through both the RcsCDB and the EnvZ/OmpR phosphorelay systems and that only the glucose backbone of OPGs is involved in virulence.

## Results

### ABF-0020261 (opgC) encodes the *O-*succinyl-transferase of the Osmoregulated Periplasmic Glucans (OPGs)

OpgC, annotated as a membrane acyl-transferase, was first identified in the non-pathogen bacteria *E. coli* K12^22^ and then in *Rhodobacter sphaeroides*^23^. In our attempt to identify the *opgC* gene in *Dickeya dadantii* (opgC_*DD*_), we first looked unsuccessfully for an homologous gene of *opgC* from *E. coli* (*opgC*_*EC*_) or *R. sphaeroides* (*opgC*_*RS*_) in the *D. dadantii* genome. We then looked for a gene encoding a protein with the two main characteristics found in the OpgC of *E. coli* and *R. sphaeroides, i.e*. 10 transmembrane segments and an acyl-transferase domain. We found 72 genes with an acyl-transferase domain, including 4 genes with 9 or 10 transmembrane domains (Table S1). These 4 genes were inactivated and OPGs of each of them were extracted. The presence of succinate on OPGs was analyzed by direct quantification of succinate after desubstitution by alkaline treatment of OPGs, then confirmed by mass spectrometry on native OPGs (see Experimental procedures). As expected for the wild-type strain (EC3937), no succinic acid could be detected in low osmolarity medium, while a total of 21.58 ± 3 μg of succinic acid per mg of glucose was found on OPGs at high osmolarity. Three mutants, ABF-0020770 (NFB7306), ABF-0016719 (NFB7307), and ABF-0018319 (NFB7435), displayed a similar profile to the wild-type, respectively 23.38 ± 7.63, 28.5 ± 5.1 and 24.43 ± 3.1 μg of succinic acid per mg of glucose at high osmolarity. In the last mutant, ABF-0020261 (NFB7305), no succinic acid could be detected regardless of the osmolarity. To confirm that the inactivation of the ABF-0020261 gene provokes the lost of succinyl substitution of OPGs, a single ectopic copy of the ABF-0020261 wild-type gene was introduced by Tn*5* transposon into the mutant (NFB7305). The complemented strain (NFB7326) restored succinyl substitution of OPGs by succinyl residues only at high-osmolarity: 24.6 ± 2.54 μg of succinic acid per mg of glucose.

**Figure 1:**
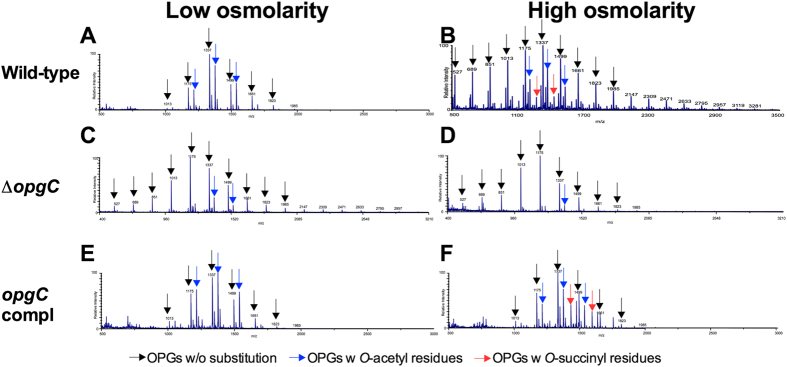
Mass spectra of purified *OPGs*. MALDI mass spectra acquired in positive ion mode on purified *OPGs* extracted from the wild type strain (**A,B**), the *opgC* strain (**C,D**) and the complemented strain (**E,F**) at low (**A,C,E**) and high (**B,D,F**) osmolarities.

The extracted OPGs from the wild-type (EC3937), ABF-0020261 mutant (NFB7305) and the complemented strains (NFB7326) were analyzed by mass spectrometry at low and high osmolarity (Fig. 1). In the mass spectra of extracted OPGs from the wild-type, OPGs with 3-12 residues of glucose were observed. This was clearly seen in mass spectra with the ten [M+Na]+ molecular ions at m/z 527, 689, 851, 1013, 1175, 1337, 1499, 1661, 1823, 1985 (black arrow, Fig. 1A,B). For several molecular ions and regardless of the osmolarity, an increment of 42 Da was observed (blue arrow, Fig. 1A,B) and corresponds to the substitution of OPGs by *O*-acetyl residues. Only for the high osmolarity, an increment of 100 Da was observed (red arrow, Fig. 1B); this corresponds to the substitution of OPGs by *O-*succinyl residues. In the mass spectra of the OPG extracted from the ABF-0020261 mutant, OPGs with only *O*-acetyl residues were observed, regardless of the osmolarity (Fig. 1C,D), confirming the loss of succinylation of OPGs. The complemented strain displayed mass spectra similar to the wild-type strain, *i.e.* OPGs with *O*-acetyl residues at low and high osmolarity and *O*-succinyl residues only at high osmolarity (Fig. 1E,F).

The ABF-0020261 gene encodes a membrane protein with an acyl-transferase required for the succinylation of the OPGs and was named *opgC*.

### *opgC* is regulated by the variation of the osmolarity

Dependence of succinyl substitution toward osmolarity led us to study the relationship between osmolarity, succinyl residues on OPGs and expression of this gene.

**Figure 2:**
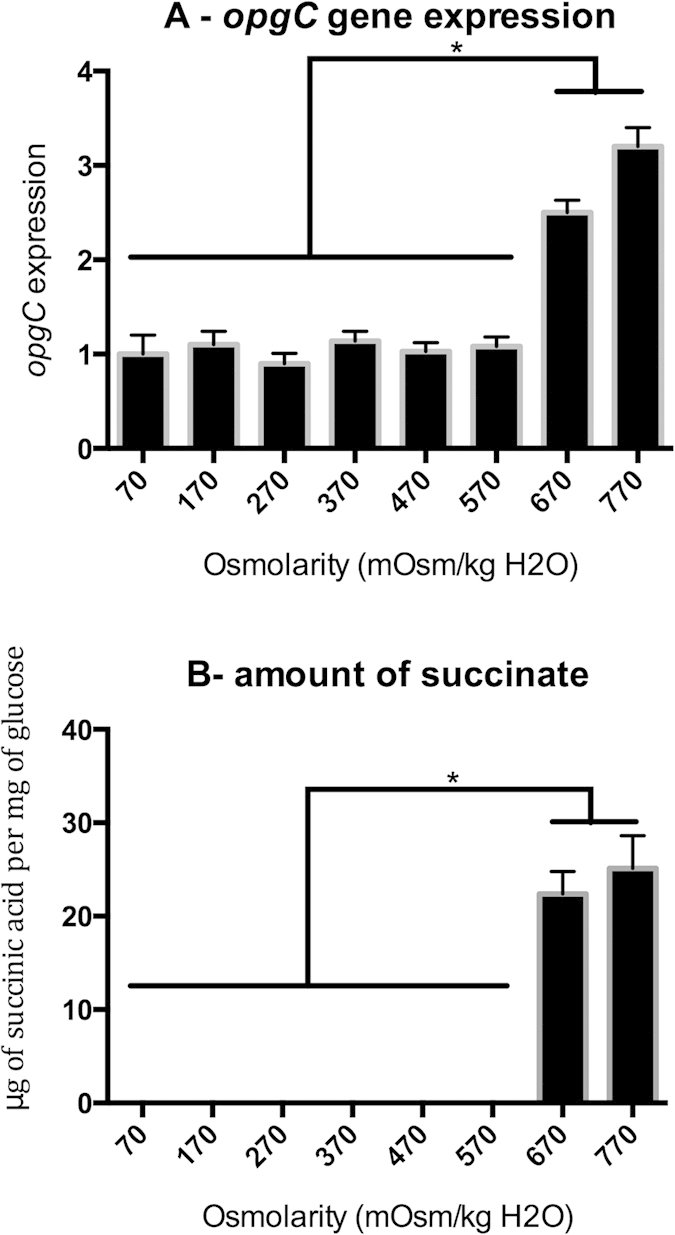
*opgC* expression level and amount of succinyl residues on purified *OPGs* in the wild-type at various osmolarities. (**A**) For the expression of the *opgC*::uidA gene, bacteria were grown to the mid-log phase and lysed by sonication. β-glucuronidase activity was measured with PNPU as a substrate. Specific activity was expressed as the change in OD405 per minute and per milligram of protein. Results are the average of three independent experiments. (**B**) For the quantification of the amount of succinyl residues, bacteria were grown overnight and *OPGs* extracted. Succinic acid content was determined with a succinic acid kit.

We started by measuring the expression of the *opgC-uidA* fusion gene and the amount of *O-*succinyl residues on the OPGs (Fig. 2). The wild-type strain was grown from 70 to 770 mosM until mid-log phase and the *opgC* level expression was measured (Fig. 2A). Until medium osmolarity, (from 70 to 570 mosM), the level of expression remained similar and low. Since no *O-*succinyl residue was detected on OPGs from 70 to 570 mosM, this low level of expression of *opgC* was insufficient for the transfer of succinyl residues on OPGs (Fig. 2B). The expression of the *opgC* gene increased abruptly 3-fold at high osmolarity (both at 670 and 770 mosM) (Fig. 2A). This increased level of expression was correlated with a substitution of OPGs by *O*-succinyl residues, the amounts being similar at both osmolarities: 22.4 ± 2.4 and 25.10 ± 3.5 μg of succinic acid per mg of glucose of OPGs. The absence or presence of *O-*succinyl residues on the OPGs was confirmed by mass spectrometry for extracted OPGs from each osmolarity (data not shown). Thus, the succinylation of the OPGs responds in a simple manner by an On/Off system, *i.e.* presence or absence of succinyl residues.

### The EnvZ-OmpR and RcsCDB phosphorelays system control the *opgC* gene

In *D. dadantii*, perception of osmolarity is mainly sensed by the EnvZ-OmpR and RcsCDB phosphorelay systems^24^. We speculated that the *O-*succinylation of the OPGs is regulated through one or both of these phosphorelay systems.

**Figure 3:**
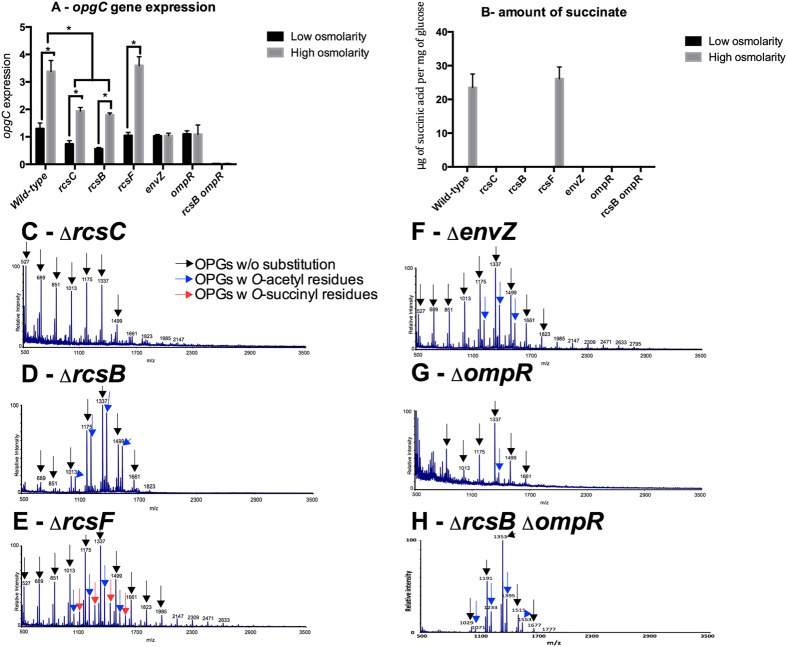
*opgC* expression level, amount of succinyl residues on purified *OPGs* and mass spectra of purified *OPGs* in the wild-type, *rcsC*, *rcsB*, *rcsF*, *envZ*, *ompR* and *rcsB ompR* mutants. (**A**) For the expression of the *opgC*::uidA gene, bacteria were grown to the mid-log phase and lysed by sonication. β-glucuronidase activity was measured with PNPU as a substrate. Specific activity was expressed as the change in OD405 per minute and per milligram of protein. Results are the average of three independent experiments. (**B**) For the quantification of the amount of succinyl residues, bacteria were grown overnight and *OPGs* extracted. Succinic acid content was determined with a succinic acid kit. (**C–H**) MALDI mass spectra acquired in positive ion mode on purified *OPGs* on various mutants.

To test this hypothesis, the *opgC* expression level was measured at low and high osmolarity in single *rcsC*, *rcsB*, *rcsF*, *envZ, ompR* mutant strains and in the double *ompR rcsB* mutant strain (Fig. 3). Expression of *opgC* in the *rcsF* mutant strain is similar to the expression observed for the wild-type strain for each osmolarity, and succinyl residues were measured at 23.4 ± 4 μg of succinic acid per mg of glucose and seen in mass spectra from OPGs extracted at high osmolarity from the *rcsF* mutant strain (Fig. 3A,B and F). In the *rcsC* and *rcsB* single mutant strains, *opgC* expression is reduced two-fold regardless of the osmolarity, indicating that regulation by osmolarity still occurs. Induction of the *opgC* gene in both mutant strains remains too low to have on effect on the OPGs substitution since no *O*-succinyl residue could be observed by mass spectra or measured on OPGs extracted from these mutant strains at any osmolarity tested (Fig. 3B,D and E). In the *envZ* and *ompR* single mutant strains, the *opgC* expression was not induced at high osmolarity and remains similar to the level observed at low osmolarity. As expected, no succinyl residue could be observed by mass spectra nor measured from OPGs extracted from these mutant strains at any osmolarity (Fig. 3A,B,G and H). In the *rcsB ompR* double mutant strain, there was no *opgC* expression and no succinyl residue could be observed by mass spectra nor measured from OPGs extracted from these mutant strains at any osmolarity (Fig. 3A,B,G and H).

These data indicate that the EnvZ-OmpR and RcsCDB phosphorelay systems are both required in the regulation of the *O-*succinylation of the OPGs. The EnvZ-OmpR phosphorelay system is involved in the induction of the system. The RcsCDB phosphorelay system is most likely involved in the enhancing of *opgC* expression in an RcsF-independent manner.

### OmpR and RcsB response regulator bind on the *opgC* promoter

**Figure 4:**
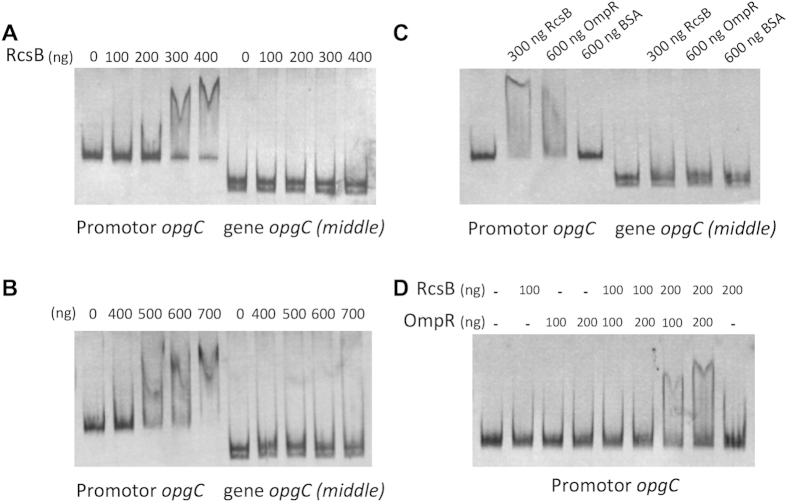
EMSA assay for RcsB, OmpR, and a mix of both proteins binding on *opgC* promoter region. Various concentration of RcsB (**A**), OmpR (**B**), BSA or mix of both proteins (**D**) were incubated with the *opgC* promoter region. The BSA (**C**) and a part of a part of the *opgC* (**A–C**) coding sequence (middle) were used as negative controls.

To know whether OmpR and RcsB regulation is achieved by a direct binding of both the transcriptional regulators on the *opgC* promoter, we performed an electrophoretic mobility shift assay (Fig. 4). After purification of the OmpR and the RcsB regulator proteins, we found that both proteins were able to bind separately on the *opgC* promoter as shown by the gel shift of the DNA probe at a minimal concentration of 500 ng and 300 ng for OmpR and RcsB respectively. Both proteins were able to bind together to this promoter, which significantly reduced both minimal concentrations to 100 ng and 200 ng for OmpR and RcsB, respectively. When the middle of the *opgC* coding sequence was used as a negative DNA control, neither OmpR nor RcsB, separately or together, bound on this DNA fragment of the *opgC* gene. When BSA was used as a negative protein control, we were not able to observe any gel shift with any part of the *opgC* gene. These results showed that OmpR and RcsB directly regulate *opgC* gene expression by binding on its promoter.

### The *opgC* genes of *E. coli* and of *D. dadantii* seem to have two different origins

**Figure 5:**
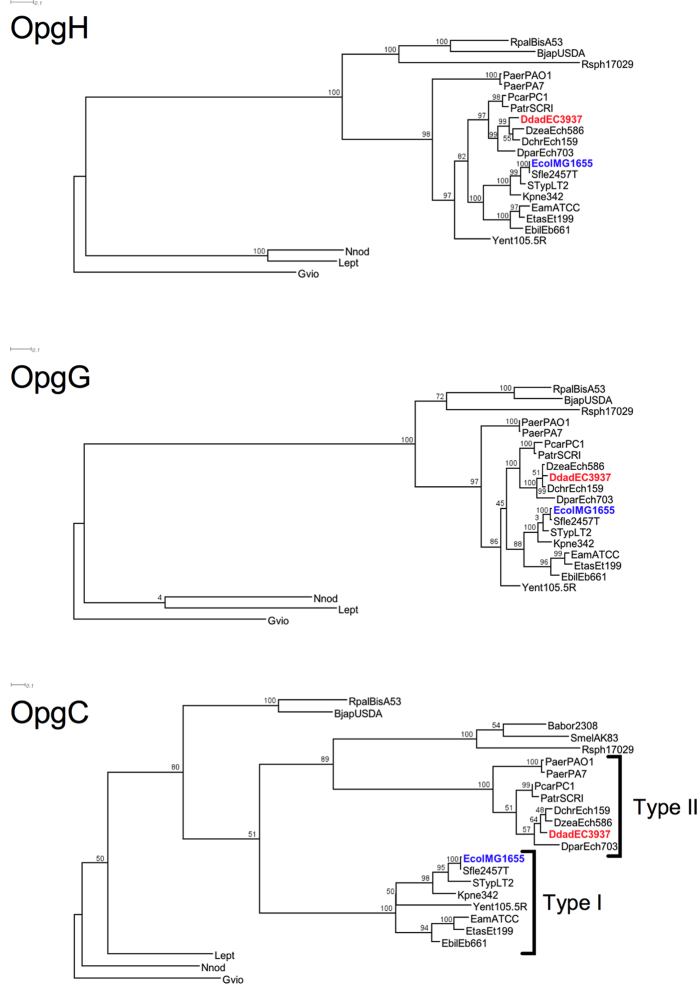
Rooted phylogenetic tree of OpgH, OpgG and *opgC* based on the maximum likelihood. Numbers on knot are bootstrap confidence levels. *Gloeobacterer* violaceus, *Leptolyngbya sp.* PCC 7375, *Leptolyngbya Nodulosa* were used as outgroup and only *Gloeobacterer* violaceus was used to root the tree. Full species names are as follows: *Bradyrhizobium japonicum* USDA 6, *Rhodopseudomonas palustris* BisA53, *Pseudomonas aeruginosa* PA7 and PAO1, *Dickeya chrysanthemi* Ech1591, *Dickeya zeae* Ech586, *Dickeya dadantii* EC3937 (in red), *Dickeya paradisiaca* Ech703, *Pectobacterium carotovorum carotovorum* PC1, *Pectobacterium atrosepticum* SCRI1043, *Klebsiella pneumoniae* 342, *Shigella flexneri* 2a str. 2457T, *Escherichia coli* K-12 substr. MG1655 (in blue), *Salmonella enterica enterica serovar Typhimurium* str. LT2, *Erwinia billingiae* Eb661, *Erwinia tasmaniensis* Et1/99, Erwinia amylovora ATCC 49946, *yersinia enterocolitica palearctica* 105.5R, *Rhodobacter sphaeroides* ATCC 17029, *Brucella melitensis* biovar *Abortus* 2308 and *Sinorhizobium meliloti* AK83. All amino acid sequences are from NCBI database.

**Figure 6:**
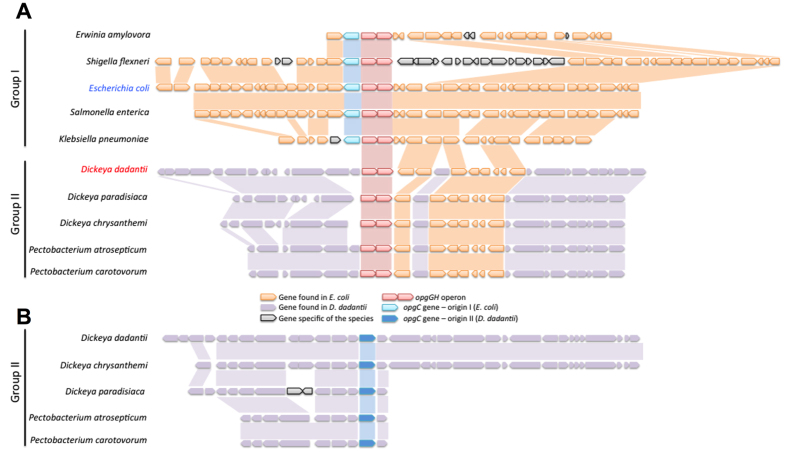
Syntheny analysis showing the genomic organization of orthologous of the opgGH operon or/and *opgC* gene. The group I of bacteria has opgGH operon and *opgC* gene link (**A**). The group II of bacteria has opgGH operon and the *opgC* gene separate on the genome (**A,B**). A macrosyntheny can be observed for the group I down the opgGH operon and in both side of the *opgC* gene for the group II.

We were wondering why we were not able to find the *opgC* gene of *D. dadantii* (*opgC*_*DD*_) by direct BLAST of the *opgC* gene of *E. coli* (*opgC*_*EC*_). We started by analyzing the identities and the similarities between the amino acid sequences by using EMBOSS Needle^25^. Only 19.5% identities and 32.5% similarities between OpgC_DD_ and OpgC_EC_ were found. We extended our study by a phylogenetic analysis. Three phylogenic trees of OpgG, OpgH (the two proteins required for backbone synthesis) and OpgC, obtained by the maximum-parsimony method, were constructed with OpgC, identified by homology with OpgC_EC_ or OpgC_DD_ (Fig. 5). Three cellulose synthases from cyanobacteria were used as an outgroup for OpgG and OpgH tree. Three transmembrane acyl-transferases from cyanobacteria were used as an outgroup for OpgC. Both OpgH and OpgG phylogenetic trees displayed a similar organization, which closely follows the organization of the tree based on the 16S RNA. In contrast, the OpgC phylogenetic tree revealed two major phylogenetic groups of OpgC: group I and group II (Fig. 5). Group I is composed of *E. coli* K12, four zoopathogenic bacteria (*Shigella flexneri*, *Salmonella enterica* pv. Typhimurium, *Klebsiella pneumoniae*, *Yersinia enterocolitica*) and three phytopathogenic bacteria (*Erwinia billingiae*, *Erwinia tasmaniensis*, *Erwinia amylovora*). Group II is composed of *D. dadantii*, five phytopathogenic bacteria (*Dickeya chrysanthemi*, *Dickeya zeae*, *Dickeya paradisiaca*, *Pectobacterium carotovorum*, *Pectobacterium atrosepticum*). Seven bacteria (*Pseudomonas aeruginosa*, *Bradyrhizobium japonicum*, *Rhodopseudomonas palustris, Rhodobacter sphaeroides*, *Brucella melitensis* biovar *Abortus* and *Sinorhizobium meliloti*) didn’t belong in either the group I or the group II. We analyzed the synteny of the *opgC* gene in the bacteria used for the phylogenetic tree. Two genetic organizations were identified (Fig. 6). In group I, the *opgC* gene was located downstream of the *opgGH* operon. Upstream of the *opgGH* operon, the synteny was conserved on 18 genes. Group II shared the same macrosyntheny from the *opgGH* operon and the upstream area with group I. The *opgC* gene is not closely related to the *opgGH* operon, as observed in group I. In group II, the areas upstream and downstream of the *opgC* gene displayed a conserved macrosynteny on 13 genes. The seven bacteria not included in group I or group II did not share any common genetic organization.

The phylograms and the synteny showed two major clusters, strongly suggesting two different origins for the *O*-succinyl transferase gene among the strains.

### A functional complementation occurs between *opgC* of *E. coli* and *opgC* of *D. dadantii*

As *opgC*_EC_ and *opgC*_DD_ seem to have two different origins, we assayed a functional complementation between *opgC*_*EC*_ and *opgC*_*DD*_. We constructed various strains in *E. coli* and *D. dadantii* with plasmids containing the *opgC*_*EC*_ gene (pNF418) or the *opgC*_*DD*_ gene (pNFW412). All the strains were grown at low and high osmolarity. The OPGs were extracted and analyzed by mass spectrometry. In the *D. dadantii* strain with *opgC*_*DD*_, the OPGs were substituted by *O*-succinyl residues only at high osmolarity as seen in the wild-type strain. In the *D. dadantii* strain with *opgC*_*EC*_, the OPGs were substituted by *O-*succinyl residues regardless of the osmolarity, as seen in the *E. coli* wild-type strain. In *E. coli* strains, whatever the combination of the gene or the osmolarity, the OPGs were always substituted by *O-*succinyl residues. The regulation of *opgC* expression is transcriptionally regulated by osmolarity in *D. dadantii*, while *E. coli* have no osmotic regulation of the *O-*succinyl transferase gene.

### Succinylated OPGs are not involved in the virulence

**Figure 7:**
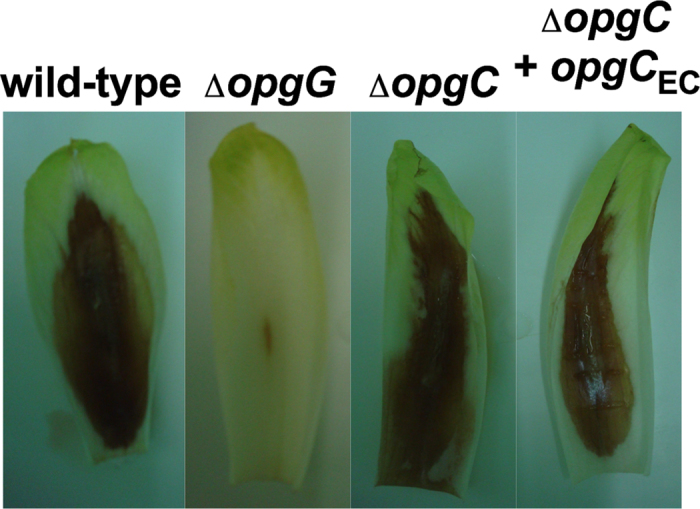
Pathogenicity of the wild-type, opgG, *opgC* mutants and the *opgC* mutant complemented with the *opgC* from *E. coli* (*opgC*_*EC*_). Chicory leaves were scarified and inoculated with 107 bacteria. Intensirty of the disease was estimated after 2 days.

OPGs are clearly virulence factors in *D. dadantii*^13^,^14^. Previously, we demonstrated that the OPGs concentration directly affects the activation level of the RcsCDB phosphorelay system. Furthermore, OPGs are synthesized at a high level to repress the RcsCDB phosphorelay system, which in turn avoids the repression of the pectinase genes set through the Rsm system, resulting in full virulence throughout the entire infectious process^26^. To figure out the role of the *O-*succinylation of the OPGs in virulence, we assayed the impact on virulence of the *opgC* strain. Chicory leaves were inoculated with the wild-type strain and *opgG* and *opgC* single mutant strains. Virulence severity was observed 2 days post-inoculation (Fig. 7). As expected, the wild-type showed the visible symptoms of the soft rot disease at 2 days post-inoculation. The *opgG* mutant strain showed no symptoms. The *opgC* strain showed the same phenotype as the wild-type.

This result was not surprising since we previously showed that *D. dadantii* synthesizes OPGs without *O-*succinyl residues throughout the infectious cycle^26^. On the contrary, the constant presence of *O-*succinyl residues on the OPGs could affect virulence. To test this hypothesis, we used the *opgC*_*DD*_ mutant strain of *D. dadantii* with the *opgC*_*EC*_ gene, which constitutively succinylated OPGs in *D. dadantii* (see preceding paragraph). Bacteria were inoculated into chicory leaves and the development of the disease was observed 2 days post-inoculation (Fig. 7). The leaves showed a similar development of the soft rot disease. OPGs extracted during infection into chicory leaves from *D. dadantii* with the *opgC*_*EC*_ gene synthetized a similar amount of OPGs succinated as the wild-type strain at high osmolarity (data not shown). Taken together, these results strongly suggest that only the backbone of the OPGs has a role in virulence.

## Discussion

In a previous paper, our lab showed that succinylation of OPGs in *D. dadantii* was osmodependent^11^. Here, we characterize the *opgC* gene responsible for the succinylation of OPGs in *D. dadantii* and decipher the mechanism by which the regulation of *opgC* occurs.

**Figure 8:**
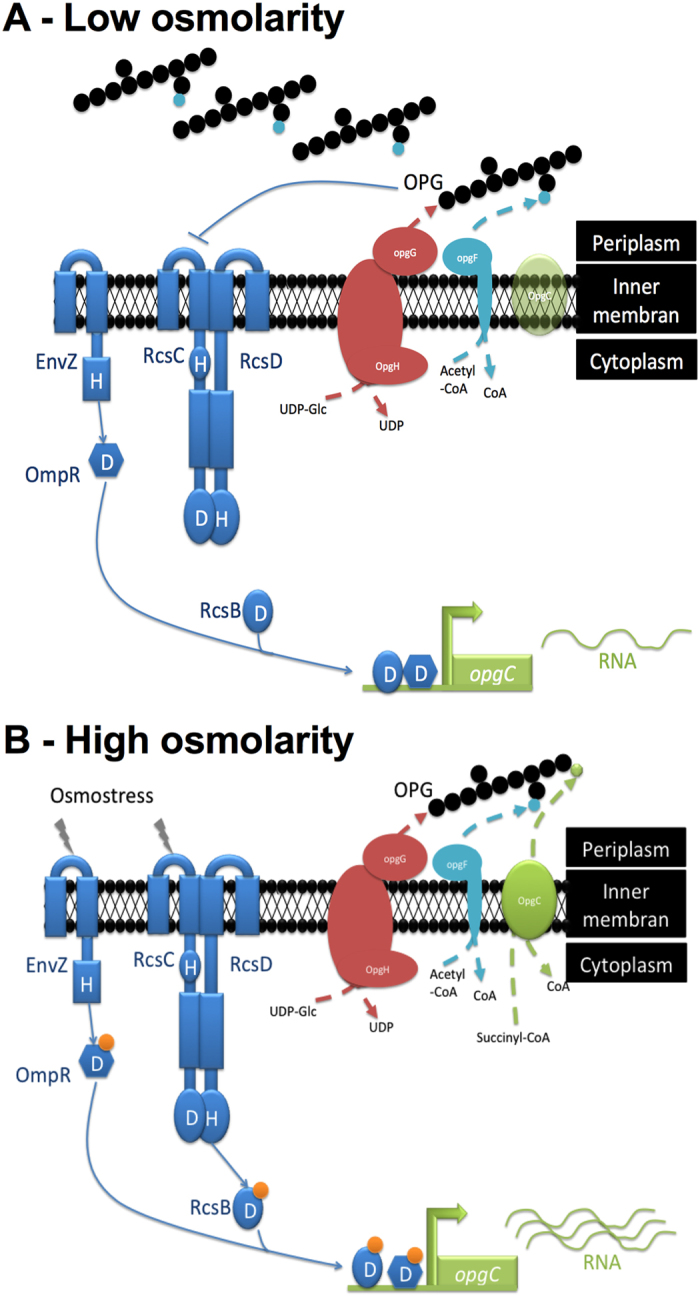
Working model of the *opgC* regulation. (**A**) At low osmolarity, both EnvZ/OmpR and RcsCDB phosphorelays aren’t activated. Binding of non-phosphorylated response regulators of both systems on *opgC* promoter allow just a basal *opgC* expression too low for succinyl substitution on *OPGs*. (**B**) At high osmolarity, both EnvZ/OmpR and RcsCDB phosphorelays are activated. Binding of phosphorylated response regulators from both systems on *opgC* promoter increased the *opgC* level expression to a level allowing succinyl substitution on *OPGs*.

In this study, we identified by *in silico* analysis the *opgC* gene (ABF-0020261). A direct search based on the *opgC* from *E. coli* was unsuccessful. Indeed we showed that the *opgC* gene from *E. coli* and *opgC* from *D. dadantii* seem to have two different origins. However, major structural features of the protein were conserved (Fig. 5). This idea of two different origins was strengthened by the analysis of their respective genomic organizations. *D. dadantii* and *E. coli* do not share the same organization (Fig. 6). One additional difference is that *opgC* of *E. coli* is constitutively expressed^22^. Interestingly, in *D. dadanti*, *opgC* is included in the *narXL* operon. This operon is known to be involved in the regulation of metabolism^27^. However, the inactivation of the *narXL* genes had no effect on OPGs synthesis or on its substitutions^26^. *opgC* gene expression is increased when the external osmolarity increases and occurrs through the EnvZ-OmpR and the RcsCDB phosphorelay systems (Figs 2, 3, 8). No expression of *opgC* was observed when these two phosphorelays were simultaneously inactivated, while a basal expression was observed when only one of the two phosphorelay was inactivated, regardless of the osmolarity (Fig. 3). Both regulators OmpR and RcsB bind separately the *opgC* regulatory DNA region (Fig. 4). However, the required amount of both proteins for binding severely decreased when added together. These two systems are necessary and sufficient for full expression and regulation of *opgC*. Both regulators are able to activate the expression of the *opgC* gene to a lower level in medium or low osmolarity although without succinylation of OPGs. Thus, succinylation of OPGs in *D. dadantii* appears to be an all-or–nothing phenomenon: succinylation on OPGs occurs only when OpgC reaches the adequate level (*i.e.* high osmolarity). Lowering this level, at least to 60% (see rcsB and rcsC mutant strains in Fig. 3), led the synthesis of OPGs completely devoid of succinyl residue (Fig. 3). Expression results show that the EnvZ-OmpR system is directly involved in the induction of the *opgC* gene by high osmolarity while RcsCDB is more likely involved in the enhancing of *opgC* gene expression. Interestingly, we showed previously that RcsCDB system activation is decreased when the amount of OPGs increased (*i.e*. in medium of low osmolarity)^14^,^15^. At high osmolarity, the concentration of the OPGs is low and in this way the RcsCDB system is activated^14^, which in turn enhances the expression level of the *opgC* gene (Figs 3 and 8A). The role of this feedback loop between the substitution of the OPGs and the activation of the RcsCDB system remains unclear but strengthens the relationship between them.

Osmoregulated periplasmic glucans are known to be an important virulence factor for many Gram-negative bacteria^8^. It was previously shown that OPGs are not substituted by succinyl residue during the infection cycle and this absence may be of importance for full virulence^14^. Thus, the normal virulence of strains synthesizing non succinylated OPGs was logical. The normal virulence of strains synthesizing OPGs constitutively succinylated indicates that the absence of *O*-succinyl substitutions on OPGs is not a prerequisite for virulence. The origin of the succinyl is probably the same in *D. dadantii* and in *E. coli* (succinyl CoA) since proteins show strong common structural features. Increased diversion of this succinyl CoA from the TCA cycle may be an additional signal to help perception of high osmolarity in *D. dadantii* relieving the RcsCDB and/or EnvZ-OmpR signals in an unknown mechanism. This additional signal cannot be used by *E. coli* even though it encounters the same kind of environmental variation.

The biological role of succinylated glucans under high osmolarity conditions remains unclear. At least, two hypothesis could be proposed. The succinylation of the OPGs gives an acidic character on OPGs synthesized. The presence of positive charge could bind free anions from the outside directly in the periplasm and avoid that too many anions arrived in the cytoplasm. In this case, the succinylated OPGs will play a role of osmoprotective molecules. We can also hypothesized that if the substrate is the succinyl CoA, used succinyl CoA could affect directly the TCA cycle by increasing or decreasing the production of ATP.

The nature of substitutions of OPGs depends on the species considered^9^. In addition, this work highlights the differences in regulation of the same substituent depending on the species. Thus, substituents of OPGs may be considered as an intrinsic property playing a biological role, eventually defined by the lifestyle of bacteria. In *Brucella abortus*^44^, the succinylated OPGs are required under hypo-osmotic condition but not during the virulence. However, no role had been assigned to any substituent in other bacterial species.

## Methods

### Bacterial strains, media and growth conditions

Bacterial strains are described in Table 1. Bacteria were grown at 30 °C for *D. dadantii* or at 37 °C for *E. coli* in lysogeny broth (LB)^28^, or in minimal medium LOS (4 g of casein hydrolysate, 0.5 mg of FeSO_4_, 18 mg of MgCl_2_, 200 mg of (NH_4_)_2_SO_4_ and 175 mg of K_2_HPO_4_ per liter, pH 7.2) supplemented with a carbon source at a concentration of 2 g.l^−1^ 29. Solid media were obtained by adding agar at 15 g.l^−1^.

Antibiotics in media were used at the following concentrations: kanamycin, 25 μg.ml^−1^; chloramphenicol, 25 μg.ml^−1^ spectinomycin 50 μg.ml^−1^ and gentamycin, 2 μg.ml^−1^.

Osmolality of growth media (mosM) was measured with vapor pressure osmometer (model 3320, Advanced Instruments, Inc., MA, USA).

### Construction of the *opgC, rcsF, envZ and ompR* mutations

In order to inactivate *opgC*, the *opgC* gene was amplified by PCR (opgCml-F: GCAGTCTGGGTGGTGCAAG and opgCml-R: ATGTCAGATAAAACAGTGGGTG) and cloned into the pCR2.1 plasmid (topo TA cloning kit, Life Technologies). A chloramphenicol DNA cassette, obtained by digestion of pNFCml^15^ by EcoRV was introduced into the resulting plasmid, digested by HincII (pNFW414).

In order to inactivate *rcsF*, a DNA fragment containing this gene was generated by PCR (rcsFSma-F: AACCCGGGATCCTGCTGGTGGTTCTGGTTTATC and rcsFXba-R: AATCTAGACTGCGCCAGCACCTGGCTGATC) and cloned into the pCR2.1 plasmid (topo TA cloning kit, Life Technologies). A chloramphenicol DNA cassette, obtained by digestion of pNFCml^15^ by EcoRV was introduced into the resulting plasmid, digested by AgeI and blunt-ended by the Klenow fragment of the DNA polymerase (Biolabs) (pNFW184).

In order to inactivate *ompR*, a DNA fragment containing this gene was amplified by PCR (ompRKpn-F: AGGTACCGCAAACGCAGCAGGCGCTAC and ompRKpn-R: AGGTACCCGCGTTGAGGTCGGCCACTT) and cloned into the pCR2.1 plasmid (topo TA cloning kit, Life Technologies). A gentamycin DNA cassette, generated by PCR and digested by HpaI^14^, was introduced into the resulting plasmid, digested by AfeI (pNFW457).

In order to inactivate envZ, a DNA fragment containing this gene was amplified by PCR (envZ-F: GCGAATCCTTCCATCTGATC and envZ-R: GCTGCTGGAAAACGTGACC) and cloned into the pJET1.2 (cloneJET PCR cloning kit, Life Technologies) A gentamycin DNA cassette^14^, was introduced into the resulting plasmid, digested by SfoI (pNFW515).

After electroporation of the four resulting plasmids, the mutations were integrated into the *D. dadantii* chromosome by marker exchange recombination in the presence of the appropriate antibiotic after successive cultures in low phosphate medium^30^.

### Cloning of the *opgC* from *D. dadantii* and *E. coli*

To clone the *opgC* gene from *D. dadantii*, the opgC DNA fragment was amplified by PCR (opgC-F: GCTCTAGAGCACCGCCACGCCGATGTC and opgC-R: GGGGTACCGTTCAGACGATGCATCGATG), digested by XbaI and KpnI and introduced into pUC18Not, digested by the same enzymes (pNFW413).

To obtain the pUTmini-Tn5-Sp^31^ containing *opgC*_*DD*_, pNFW413 was digested by NotI and the released *opgC*_*DD*_ DNA fragment was cloned into the pUTmini-Tn5-Sp plasmid digested by the same enzyme. This plasmid was introduced into *D. dadantii* by conjugation.

To obtain the pUTmini-Tn5-Sp containing *opgC*_*EC*_, pNF418^22^ was digested by PstI and inserted into pUC18Not digested by the same enzyme. This last plasmid was digested by NotI and the released *opgC*_EC_ DNA fragment was cloned into the pUTmini-Tn5-Sp plasmid digested by the same enzyme. This plasmid was introduced into *D. dadantii* by conjugation.

### Construction of the *opgC::uidA* gene fusion

To construct the *opgC::uidA* gene fusion, the coding DNA sequence of the *uidA* gene was amplified by PCR (GusSph-F: CACAGCATGCATTCCGGACCAGTATTATTATC and GusHind-R: CACAAAGCTTATCCGCTCACAATTCCAC) from pCRS548^32^, digested by SphI and HindIII and cloned into the pUC18not plasmid digested by the same enzymes. The *opgC* regulatory DNA region was amplified by PCR (promXba-F: CACATCTAGACGATGGTTATCTGCTCAAGG and promSph: CACAGCATGCTCATCCAAGCAGAAACCGGAG), digested by XbaI and SphI and cloned into the pUC18Not-*uidA* plasmid digested by the same enzymes.

To obtain the pUTmini-Tn5-Sp containing *opgC::uidA*, this last plasmid was digested by NotI and the released *opgC::uidA* DNA fragment was cloned into the pUTmini-Tn5-Sp plasmid digested by the same enzyme. This plasmid was introduced into *D. dadantii* by conjugation.

### Cloning, expression and purification of His-tagged RcsB and OmpR

The plasmid used to produce the recombinant His-Tagged RcsB protein was described previously (Bontemps-Gallo *et al*. 2013).

To construct the recombinant His-tagged OmpR protein, the *ompR* DNA fragment was amplified by PCR (ompR-F: CACCATGCAAGAGAATTATAAAATTCTGG and ompR-R: CCATCGAATCATGCTTTACTGCCG) and cloned into the His-tag expression vector pET100/D-Topo (Invitrogen, Life Technologies).

Both recombinant proteins were expressed in *E. coli* BL21(DE3) and purified as described previously^14^. Briefly, after growing the cell was lysed by sonication and the crude extract was transferred to a Ni-NTa column (Macherey Nagel) to purify the protein.

### Transduction, conjugation and transformation

Transformation of *E. coli* cells was carried out by the rubidium chloride technique^29^. Construction of strains was performed by transferring genes from one strain of *D. dadantii* to another by generalized transduction with phage ΦEC2 as described previously^33^. Plasmids were introduced in *D. dadantii* by conjugation or electroporation.

### Transposon mutagenesis

To allow integration of a single ectopic copy of the mini-Tn*5-Sp*-*opgC::uidA* gene fusion, mini-Tn*5-Sp*-*opgC*_*DD*_ and mini-Tn*5-Sp*-*opgC*_*EC*_, transposon mutagenesis was performed as described previously^15^. Briefly, after conjugation between an *E. coli* strain harboring the pUTmini-Tn*5-*Sp plasmid carrying the appropriate construction and a *D. dadantii* strain, Sp^r^ mutants were selected on M63 plates containing sucrose as a unique carbon source and spectinomycin.

### Determination of enzyme activities

β-glucuronidase assays were performed on crude extracts obtained from bacteria disrupted by sonication 2 × 20 s (Sonifier cell disruptor B-30, Branson, 70% duty cycle, 7 microtip limit, Hold time, continuous, appropriate probe) after growth *in vitro* (LB medium) or *in planta* and extracted from chicory leaves as described elsewhere^14^. β-glucuronidase activity was determined by spectrometric monitoring of the hydrolysis of PNPU (4-nitrophenyl- β-D-glucuronide) at 405 nm.

The protein concentration was determined by the Bradford assay with bovine serum albumin as a standard^34^.

### Pathogenicity test

Chicory leaves were inoculated as previously described^13^. Briefly, bacteria from an overnight culture in LB medium were recovered by centrifugation and diluted in physiological water. After wounding, plants were inoculated with 10^7^ bacteria and incubated in a dew chamber at 28 °C for 48 h.

### Extraction of OPGs

Bacteria were grown overnight in LOS with the indicated NaCl concentration. Bacteria were collected by centrifugation at 4 °C for 15 min at 8,000 g. Cell pellets were resuspended in distilled water and lysed with 5% trichloroacetic acid (TCA). After centrifugation at 4 °C for 15 min at 8,000 g, the supernatant was neutralized with ammonium hydroxide 10% and concentrated by rotary evaporation. The resulting material (2 ml) was then fractionated by gel filtration on a Bio-Gel P-4 column (lengh: 55 cm, diameter; 1.6 cm, Bio-Rad) equilibrated with 0.5% acetic acid. The column was eluted in the same buffer at a flow rate of 15 ml h^−1^, and fractions of 1.5 ml were collected. Presence of oligosaccharides in each fraction was determined by the colorimetrically anthrone procedure. Fractions containing OPGs were pooled and total content was determined by the same procedure^35^. For mass spectrometry analysis and succinate quantification, OPGs were subsequently desalted in water on a Bio-Gel P-2 column (lengh: 90 cm, diameters; 1.6 cm, Bio-Rad,) and fractions containing OPGs were lyophilized.

### Determination of succinate content of OPGs

After purification of the OPGs as described above, one milligram of OPGs was dissolved in 0.2 ml of 0.5 M NaOH and incubated at 100 °C for 30 min to liberate the succinyl from OPGs. Glucosidic backbones were removed by absorption on 50 mg of charcoal suspended in 0.3 ml of water, and the charcoal was then washed three times with 0.5 ml of water. The four supernatants were pooled (2 ml) and neutralized with Dowex AG 50W-X8 (Bio-Rad) on H+ form. Succinic acid content was determined with a succinic acid kit (R-Biopharm) following the manufacturer’s instruction. The limit of detection is of 2.1 mg/mg of OPGs.

### Mass spectrometry

All mass spectra were acquired on a Voyager Elite (DE-STR) reflectron time-of-flight (TOF) mass spectrometer (Perseptive Biosystems, Framingham, MA), equipped with a pulsed nitrogen laser (337 nm) and a gridless delayed extraction ion source. Oligosaccharide samples were analyzed in delayed extraction mode using an accelerating voltage of 20 kV, a pulse delay time of 200 ns and a grid voltage of 66%. Detector bias gating was used to reduce the ion current for masses below 500 Da. Between 100 and 200 scans were averaged for each mass spectrum. Oligosaccharide alditols were co-crystallized with 2,5-dihydroxybenzoic acid (DHB) as matrix [10 mg ml^−1^ of DHB in methanol/water (50/50) containing 0.1% Trifluoro Acetic acid (TFA)]. For all measurements, the “dried droplet” preparation technique was used. Typically, 1 μl of the matrix was mixed on-target with 1 μl of water-dissolved oligosaccharides and allowed to dry under an air stream. They were analyzed in positive ion mode.

### Gel shift assays

DNA fragments of about 600bp containing either the *opgC* regulatory region or middle of the *opgC* gene coding region (the control fragment) were amplified by PCR (same primer pair used for *opgC::uidA* gene fusion (see before) or opgCmid-F:AACTGACCAGCATGGGATTC) and opgCmid-R:GGGGTTGCGTTGTAGCAGG respectively). PCR products were purified using the NucleoSpin Gel and PCR Clean-up kit (Macherey-Nagel). Reaction mixtures (20 μl) containing 50 ng of each DNA fragment and various amounts of RcsB-6His and/or OmpR-6His complex in gel shift buffer (10 mM Tris [pH 7.5], 50 mM NaCl, 0.5 mM dithiothreitol [DTT], 1 mM MgCl2, and 2.5% glycerol) were incubated for 20 min at room temperature (RT) and were loaded onto a 6% acrylamide–TBE (Tris-borate EDTA) gel (89 mM Tris-borate [pH 8], 2 mM EDTA). After migration, DNA was visualized by ethidium bromide staining^36^.

### Phylogeny tree

The protein sequences of the 21 strains were aligned with CLUSTAL OMEGA^37^ with default parameters. The trees were generated using the protein maximum likelihood (Proml) method in the Phylip package (version 3.69). The node reliability of the phylogenetic trees was evaluated with 100 bootstrap replicates (seqboot, consense and retree methods of the Phylip package). The protein sequence of the strain *Gloeobacter violaceus* was used as the outgroup. Phylogenetic trees were displayed with Dendroscope (version 3.2.10).

### Statistical Analysis

For statistical analyses, Graph-prism6 software was used. Data were analyzed by paired t-test; a value of p < 0.05 was considered significant.

## Additional Information

**How to cite this article**: Bontemps-Gallo, S. *et al*. The *opgC* gene is required for OPGs succinylation and is osmoregulated through RcsCDB and EnvZ/OmpR in the phytopathogen *Dickeya dadantii. Sci. Rep.* **6**, 19619; doi: 10.1038/srep19619 (2016).

## Supplementary Material

Supplementary Information

## Figures and Tables

**Table 1:** Strain and plasmids.

Strain	Genotype/Phenotype	Reference
*D. dadantii*		
EC3937	Wild-type	Laboratory collection
NFB3682	*rcsC*::Cml	15
NFB3723	*opgG*::Cml	Laboratory collection
NFB3785	*rcsF*::Cml	This study
NFB7199	*rcsB*::Cml	15
NFB7279	*rcsB*::Gm	14
NFB7305	*opgC*::Cm	This study
NFB7326	*opgC*::Cm miniTn5Sp^R^ *opgC*_*DD*_	This study
NFB7422	*ompR*::Gm	This study
NFB7424	*envZ*::Gm	This study
NFB7459	miniTn*5*Sp^R^ *opgC*::*uidA*	This study
NFB7461	*rcsB*::Gm miniTn*5*Sp^R^ *opgC*::*uidA*	This study
NFB7464	*rcsC*::Cm miniTn*5*Sp^R^ *opgC* ::*uidA*	This study
NFB7465	*rcsF*::Cm miniTn*5*Sp^R^ *opgC*::*uidA*	This study
NFB7556	*ompR*::Gm miniTn*5*Sp^R^ *opgC*::*uidA*	This study
NFB7557	*envZ*::Gm miniTn*5*Sp^R^ *opgC* ::*uidA*	This study
NFB7616	*rcsB*::Cml *ompR*::Gm	This study
NFB7622	*rcsB*::Cml *ompR*::Gm miniTn*5*Sp^R^ *opgC*::*uidA*	This study
NFB7629	*opgC*::Cm miniTn5Sp^R^ *opgC*_*EC*_	This study
*E. coli*		
BL21(DE3)	*ompT hsdSB gal dcm*	Invitrogen
DF214	*his pgi::*Mu ∆(*zwf-edd*) *eda-1 rpsL*	22
JM83	*ara* ∆(*lac-pro*) *thi rpsL* (ϕ80 ∆*lacZ* ∆M15)	38
Top10F’	F’[*lacI*^q^Tn*10*] *mrcA* ∆(*mrr-hsd*RMS-*mcrBC*) ϕ80lacZ∆M15 ∆l*acX74 deoR nupG recA1 araD139* ∆(*ara-leu*)7697 *galU galK rpsL endA*	Invitrogen
S17I ʎPir	*recA thi pro hsdR* ʎpir RP-4-2-Tet::Mu-Kan::Tn*7*, Tmp^R^ Str^R^	39
NF702	JM83 *opgG202*::*neo*	40
NF1919	DF214 *opgC*::Tn*5*	22
Plasmids		
pNF244	pUC8 *opgGH*_*EC*_	41
pNF418	pUC8 *opgC*_*EC*_	22
pNFCml	Cml^R^	15
pCR2.1	Amp^R^	Life Technologies
pJET1.2	Amp^R^	Life Technologies
pET100/D-Topo	Amp^R^	Life Technologies
pUC18Not	Amp^R^	42
pUC18Not-*uidA*	‘*uidA*, Amp^R^	43
pUTmini-Tn*5*Sp	miniTn*5*Sp *oriR6K*, Sp^R^ Amp^R^	31
pNFW32	pUC18 *opgGH*_*DD*_	13
pNFW184	pCR2.1 *rcsF*::Cml	This study
pNFW412	pCR2.1 *opgC*_*DD*_	This study
pNFW413	pUC18-Not *opgC*	This study
pNFW414	pCR2.1 *opgC*::Cml	This study
pNFW410	pET100/D-Topo *rcsB*	13
pNFW421	pUTmini-Tn*5*-*opgC*_*DD*_	This study
pNFW444	pET100/D-Topo *ompR*	This study
pNFW457	pCR2.1 *ompR*::Gm	This study
pNFW495	pUTmini-Tn*5*-*opgC::uidA*	This study
pNFW515	pJET2.1 *envZ*::Gm	This study
pNFW547	pUTmini-Tn5-*opgC*_*EC*_	This study

## References

[b1] Glasner J. D. . Genome sequence of the plant-pathogenic bacterium *Dickeya dadantii* 3937. J Bacteriol 193, 2076–2077, doi: 10.1128/JB.01513-10 (2011). 21217001 PMC3133054

[b2] Toth I. K. . Dickeya species: an emerging problem for potato production in Europe. Plant Pathology 60, 385–399, doi: 10.1111/j.1365-3059.2011.02427.x (2011).

[b3] EPPO. Data sheets on quarantine organisms, Erwinia chrysanthemi Burkholder., 21–25 (1982).

[b4] EPPO. A1 and A2 Lists of Quarantine Pests. Specific Quarantine Requirements. EPPO/EPPO Publications Series B 92 (1988).

[b5] EPPO. Specific Quarantine Requirements. EPPO: EPPO Technical Documents 1008 (1990).

[b6] Barras F., van Gijsegem F. & Chatterjee A. K. Extracellular Enzymes and Pathogenesis of Soft-Rot Erwinia. Annual Review of Phytopathology 32, 201–234, doi: 10.1146/annurev.py.32.090194.001221 (1994).

[b7] Collmer A. & Keen N. T. The Role of Pectic Enzymes in Plant Pathogenesis. Annual Review of Phytopathology 24, 383–409, doi: 10.1146/annurev.py.24.090186.002123 (1986).

[b8] Bontemps-Gallo S. & Lacroix J. M. New Insights into the Biological Role of the Osmoregulated Periplasmic Glucans in Pathogenic and Symbiotic Bacteria. Environ Microbiol Reports, doi: 10.1111/1758-2229.12325 (2015). PMC4618058 26265506

[b9] Bohin J. P. & Lacroix J. M. In The Periplasm (American Society of Microbiology, 2007).

[b10] Lequette Y., Lanfroy E., Cogez V., Bohin J. P. & Lacroix J. M. Biosynthesis of osmoregulated periplasmic glucans in Escherichia coli: the membrane-bound and the soluble periplasmic phosphoglycerol transferases are encoded by the same gene. Microbiology 154, 476–483, doi: 10.1099/mic.0.2007/013169-0 (2008). 18227251

[b11] Cogez V., Talaga P., Lemoine J. & Bohin J. P. Osmoregulated periplasmic glucans of Erwinia chrysanthemi. J Bacteriol 183, 3127–3133, doi: 10.1128/JB.183.10.3127-3133.2001 (2001). 11325941 PMC95213

[b12] Bouchart F., Delangle A., Lemoine J., Bohin J. P. & Lacroix J. M. Proteomic analysis of a non-virulent mutant of the phytopathogenic bacterium Erwinia chrysanthemi deficient in osmoregulated periplasmic glucans: change in protein expression is not restricted to the envelope, but affects general metabolism. Microbiology 153, 760–767, doi: 10.1099/mic.0.2006/000372-0 (2007). 17322196

[b13] Page F. . Osmoregulated periplasmic glucan synthesis is required for Erwinia chrysanthemi pathogenicity. J Bacteriol 183, 3134–3141, doi: 10.1128/JB.183.10.3134-3141.2001 (2001). 11325942 PMC95214

[b14] Bontemps-Gallo S. . Concentration of osmoregulated periplasmic glucans (OPGs) modulates the activation level of the RcsCD RcsB phosphorelay in the phytopathogen bacteria *Dickeya dadantii*. Environ Microbiol 15, 881–894, doi: 10.1111/1462-2920.12054 (2013). 23253096

[b15] Bouchart F. . The virulence of a *Dickeya dadantii* 3937 mutant devoid of osmoregulated periplasmic glucans is restored by inactivation of the RcsCD-RcsB phosphorelay. J Bacteriol 192, 3484–3490, doi: 10.1128/JB.00143-10 (2010). 20418397 PMC2897653

[b16] Madec E., Bontemps-Gallo S. & Lacroix J. M. Increased phosphorylation of the RcsB regulator of the RcsCDB phosphorelay in strains of *Dickeya dadantii* devoid of osmoregulated periplasmic glucans revealed by Phos-tag gel analysis. Microbiology 160, 2763–2770, doi: 10.1099/mic.0.081273-0 (2014). 25320363

[b17] Yoshida T., Cai S. & Inouye M. Interaction of EnvZ, a sensory histidine kinase, with phosphorylated OmpR, the cognate response regulator. Mol Microbiol 46, 1283–1294 (2002). 12453215 10.1046/j.1365-2958.2002.03240.x

[b18] Condemine G. & Ghazi A. Differential regulation of two oligogalacturonate outer membrane channels, KdgN and KdgM, of *Dickeya dadantii* (Erwinia chrysanthemi). J Bacteriol 189, 5955–5962, doi: 10.1128/JB.00218-07 (2007). 17573480 PMC1952035

[b19] Carballes F., Bertrand C., Bouche J. P. & Cam K. Regulation of Escherichia coli cell division genes ftsA and ftsZ by the two-component system rcsC-rcsB. Mol Microbiol 34, 442–450 (1999). 10564486 10.1046/j.1365-2958.1999.01605.x

[b20] Clarke D. J. The Rcs phosphorelay: more than just a two-component pathway. Future Microbiol 5, 1173–1184, doi: 10.2217/fmb.10.83 (2010). 20722597

[b21] Majdalani N. & Gottesman S. The Rcs phosphorelay: a complex signal transduction system. Annu Rev Microbiol 59, 379–405, doi: 10.1146/annurev.micro.59.050405.101230 (2005). 16153174

[b22] Lacroix J. M. . The mdoC gene of Escherichia coli encodes a membrane protein that is required for succinylation of osmoregulated periplasmic glucans. J Bacteriol 181, 3626–3631 (1999). 10368134 10.1128/jb.181.12.3626-3631.1999 PMC93837

[b23] Cogez V., Gak E., Puskas A., Kaplan S. & Bohin J. P. The opgGIH and opgC genes of Rhodobacter sphaeroides form an operon that controls backbone synthesis and succinylation of osmoregulated periplasmic glucans. Eur J Biochem 269, 2473–2484 (2002). 12027885 10.1046/j.1432-1033.2002.02907.x

[b24] Clarke E. J. & Voigt C. A. Characterization of combinatorial patterns generated by multiple two-component sensors in *E. coli* that respond to many stimuli. Biotechnol Bioeng 108, 666–675, doi: 10.1002/bit.22966 (2011). 21246512 PMC3413328

[b25] McWilliam H. . Analysis Tool Web Services from the EMBL-EBI. Nucleic Acids Res 41, W597–600, doi: 10.1093/nar/gkt376 (2013). 23671338 PMC3692137

[b26] Bontemps-Gallo S. *Dickeya dadantii* : *vers la compréhension du rôle biologique des glucanes périplasmiques osmorégulés PhD thesis*, University of Lille, (2013).

[b27] Constantinidou C. . A reassessment of the FNR regulon and transcriptomic analysis of the effects of nitrate, nitrite, NarXL, and NarQP as Escherichia coli K12 adapts from aerobic to anaerobic growth. J Biol Chem 281, 4802–4815, doi: 10.1074/jbc.M512312200 (2006). 16377617

[b28] Bertani G. Lysogeny at mid-twentieth century: P1, P2, and other experimental systems. J Bacteriol 186, 595–600 (2004). 14729683 10.1128/JB.186.3.595-600.2004 PMC321500

[b29] Miller J. H. In A short course in bacterial genetics: a laboratory manual and handbook for Escherichia coli and related bacteria (Cold Spring Harbor Laboratory Press edn, 1992).

[b30] Roeder D. L. & Collmer A. Marker-exchange mutagenesis of a pectate lyase isozyme gene in Erwinia chrysanthemi. J Bacteriol 164, 51–56 (1985). 2995324 10.1128/jb.164.1.51-56.1985 PMC214209

[b31] de Lorenzo V., Herrero M., Jakubzik U. & Timmis K. N. Mini-Tn5 transposon derivatives for insertion mutagenesis, promoter probing, and chromosomal insertion of cloned DNA in gram-negative eubacteria. J Bacteriol 172, 6568–6572 (1990). 2172217 10.1128/jb.172.11.6568-6572.1990 PMC526846

[b32] Reeve W. G. . Constructs for insertional mutagenesis, transcriptional signal localization and gene regulation studies in root nodule and other bacteria. Microbiology 145 **(Pt 6)**, 1307–1316 (1999). 10411257 10.1099/13500872-145-6-1307

[b33] Resibois A., Colet M., Faelen M., Schoonejans E. & Toussaint A. phiEC2, a new generalized transducing phage of Erwinia chrysanthemi. Virology 137, 102–112 (1984). 18639822 10.1016/0042-6822(84)90013-8

[b34] Bradford M. M. A rapid and sensitive method for the quantitation of microgram quantities of protein utilizing the principle of protein-dye binding. Anal Biochem 72, 248–254 (1976). 942051 10.1016/0003-2697(76)90527-3

[b35] Spiro R. G. In Methods in Enzymology Vol. 8 (ed Victor Ginsburg Elizabeth F. Neufeld ) 3–26 (Academic Press, 1966).

[b36] Bibi-Triki S. . Functional and structural analysis of HicA3-HicB3, a novel toxin-antitoxin system of Yersinia pestis. J Bacteriol 196, 3712–3723, doi: 10.1128/JB.01932-14 (2014). 25112480 PMC4248797

[b37] Sievers F. . Fast, scalable generation of high-quality protein multiple sequence alignments using Clustal Omega. Mol Syst Biol 7, 539, doi: 10.1038/msb.2011.75 (2011). 21988835 PMC3261699

[b38] Vieira J. & Messing J. The pUC plasmids, an M13mp7-derived system for insertion mutagenesis and sequencing with synthetic universal primers. Gene 19, 259–268 (1982). 6295879 10.1016/0378-1119(82)90015-4

[b39] de Lorenzo V. & Timmis K. N. Analysis and construction of stable phenotypes in gram-negative bacteria with Tn5- and Tn10-derived minitransposons. Methods Enzymol 235, 386–405 (1994). 8057911 10.1016/0076-6879(94)35157-0

[b40] Loubens I., Debarbieux L., Bohin A., Lacroix J. M. & Bohin J. P. Homology between a genetic locus (mdoA) involved in the osmoregulated biosynthesis of periplasmic glucans in Escherichia coli and a genetic locus (hrpM) controlling pathogenicity of Pseudomonas syringae. Mol Microbiol 10, 329–340 (1993). 7934824 10.1111/j.1365-2958.1993.tb01959.x

[b41] Lacroix J. M., Tempete M., Menichi B. & Bohin J. P. Molecular cloning and expression of a locus (mdoA) implicated in the biosynthesis of membrane-derived oligosaccharides in Escherichia coli. Mol Microbiol 3, 1173–1182 (1989). 2552262 10.1111/j.1365-2958.1989.tb00267.x

[b42] Yanisch-Perron C., Vieira J. & Messing J. Improved M13 phage cloning vectors and host strains: nucleotide sequences of the M13mp18 and pUC19 vectors. Gene 33, 103–119 (1985). 2985470 10.1016/0378-1119(85)90120-9

[b43] Bontemps-Gallo S., Madec E. & Lacroix J. M. Inactivation of pecS restores the virulence of mutants devoid of osmoregulated periplasmic glucans in the phytopathogenic bacterium *Dickeya dadantii*. Microbiology 160, 766–777, doi: 10.1099/mic.0.074484-0 (2014). 24550070

[b44] Roset M. S., Ciocchini A. E., Ugalde R. A. & Inon de Iannino N. The Brucella abortus cyclic beta-1,2-glucan virulence factor is substitued with O-ester-linked succinyl residues. J. Bacteriol 188, 5001–5013, doi: 10.1128/JB.00086-06 (2006). PMC1539967 16816173

